# Shear Wave Elastography of the Skin following Radial Forearm Free Flap Surgery in Transgender Patients: Observational Study

**DOI:** 10.3390/jcm13164903

**Published:** 2024-08-20

**Authors:** Martino Guiotto, Oana Cristina Sciboz, Carmen Arquero, Luigi Schiraldi, Pietro Di Summa, Olivier Bauquis, Sébastien Durand

**Affiliations:** 1 Department of Plastic, Reconstructive and Hand Surgery, Lausanne University Hospital, 1011 Lausanne, Switzerland; martino.guiotto@chuv.ch (M.G.); luigi.schiraldi@chuv.ch (L.S.); pietro.di-summa@chuv.ch (P.D.S.); olivier.bauquis@chuv.ch (O.B.); 2 Department of Paediatric Surgery, Lausanne University Hospital, 1011 Lausanne, Switzerland; oana-cristina.sciboz@chuv.ch; 3 Department of Vascular Surgery, Lausanne University Hospital, 1011 Lausanne, Switzerland; carmen.arquero@chuv.ch

**Keywords:** shear wave elastography, ultrasound, wound healing, radial forearm free flap, transgender, Patient and Observer Scar Assessment Scale (POSAS)

## Abstract

**Background:** Ultrasound shear wave elastography (SWE) noninvasively measures the stiffness of tissue by producing and measuring tissue deformation. Scar formation, a crucial aspect of wound healing, can lead to functional and aesthetic complications when pathological. While SWE has shown promise in dermatological evaluations, its role in surgical scar assessment remains underestimated. Our study aims to investigate SWE in evaluating surgical scars at the donor site after forearm free flap surgery in transgender patients. **Methods:** After radial forearm free flap harvesting, the donor site was grafted with a split-thickness skin graft with or without interposition of Matriderm. Eleven patients were evaluated more than one year after surgery, using SWE alongside scar characteristics, sensory outcomes, and patient satisfaction surveys. **Results:** Our study revealed no significant difference in stiffness (*p* > 0.15), pigmentation (*p* = 0.32), or erythema (*p* = 0.06) between operated and non-operated sides. The interposition of Matriderm did not influence the stiffness. Patients significantly (*p* < 0.0001) reported a loss of discrimination. Patients’ subjective scar evaluation appeared in line with our quantitative and objective results. **Conclusions:** This study contributes to the evolving understanding of SWE’s role in scar assessment, highlighting its feasibility in evaluating surgical scars. However, continued research efforts are necessary to establish SWE as a reliable and objective method for surgical scar evaluation and management.

## 1. Introduction

Ultrasound elastography (UE) noninvasively measures the stiffness of the tissue by producing and measuring tissue deformation [1]. Indeed, the stiffness represents the tendency of the tissue to resist deformation induced by an applied force and is characterized by the modulus of elasticity (Young’s modulus; Pa) defined as follows: E = σ/ε, where σ is the stress (Pa), corresponding to the normalized force, and ε is the strain (unitless) which represents the deformation of the tissue due to an applied force [2]. Shear wave elastography (SWE) applies an acoustic radiation force (ARF) generated from the ultrasound transducer itself rather than a force applied manually by the examiner. The tissue displacement obtained by the ARF determines the shear waves that propagate away from the force and are tracked by multiple imaging beams, thus providing a measurement of shear wave velocity (SWV). This system is less user-dependent compared to traditional ultrasound imaging [3,4]. With the hypothesis of an isotropic, homogeneous, incompressible medium, SWS is measured and converted to Young’s modulus (E) using the following equation: E = 3 ρ c^2^, where ρ is the density and c the shear wave speed. Shear wave elastography (SWE) generates real-time monitoring of shear waves in 2D and quantitative elastograms superimposed on a B-mode image [5]. This technique was introduced in the early 1990s in vitro [6] and then expanded into clinical practice in the fields of senology, hepatology, thyroid diseases, prostatic diseases, musculoskeletal conditions [7,8,9,10,11], wound healing, and dermatology, exploring, for example, SWE application in systemic sclerosis, keloids, hypertrophic scars, and skin tumors [12,13,14,15].

Scar formation is a physiological evolution of the wound healing process after a skin trauma or surgery. When this mechanism is disrupted by intrinsic or extrinsic factors, a pathological scar can result, leading to functional and aesthetic disruptions [16]. This self-sustained fibrotic cycle underpinning pathologic scar formation is clinically silent for a long time before appearing palpable and visible, remaining active for many years after injury. Indeed, considering pathological scarring as a chronic process, where the initial stimulus to heal is perpetuated, carcinogenic transformation could take place over time [17]. Therefore, adequate prevention, early diagnosis, and treatment are advocated. Moreover, when pathological scars are compared with normal tissue in terms of appearance and mechanical properties, they are characterized by increased stiffness and thickness with reduced extensibility and elasticity, leading to chronic pain, psychological distress, and functional movement limitations in the anatomical areas involved [18].

From a clinical perspective, early intervention on an immature scar is recognized as more effective than late treatment of an old, mature scar. However, the current clinical assessment of scars relies on inadequate tools and relies mainly on medical examination and doctor’s experience, which often leads to delays in diagnosis and ineffective treatment [16]. Therefore, new methods for evaluating the wound healing process are advocated to allow an early diagnosis of pathological scars, effective preventive strategies, and early treatment, thus avoiding patient discomfort or late and more invasive interventions.

Finally, an objective and reproducible scar assessment tool for pathological scarring is also required for developing and comparing novel treatments in order to evaluate their efficacy and safety and to compare them with other already approved ones. The role of SWE in surgical scar assessment remains underestimated.

The aim of our study is to explore the application of SWE in clinical surgical scar assessment at the donor site (skin grafted) after forearm free flap surgery in transgender patients.

## 2. Materials and Methods

### 2.1. Subjects

**Figure 1:**
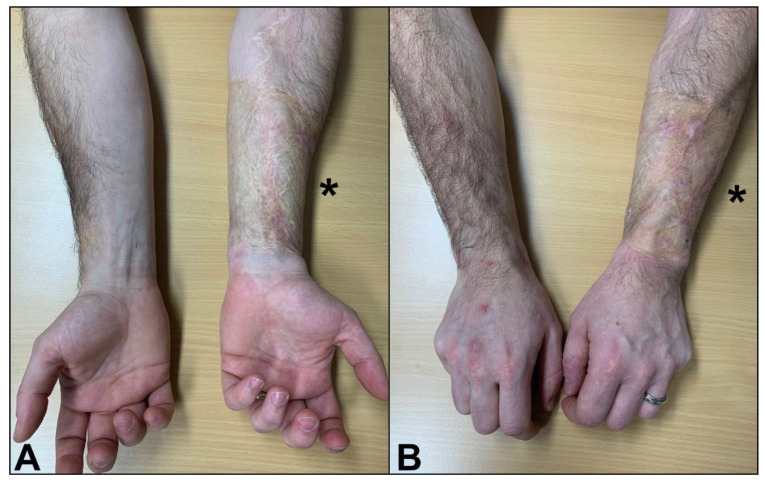
Clinical outcome: anterior (**A**) and posterior (**B**) aspects of the skin-grafted donor site post-radial forearm free flap. *: donor site.

Written consent was obtained from all patients. This retrospective chart review study involving human participants was conducted in accordance with the ethical standards of the institutional and national research committee and with the 1964 Helsinki Declaration. This study received approval from the local ethics committee (CER-VD, BASEC-ID 2022-02162). All patients who underwent to radial forearm free flap for phalloplasty, performed by the same surgeon (O.B.) at our institution between 1 January 2012 and 30 April 2022, were included. The examination was performed a minimum of one year (mean: 39 months) after surgery (Figure 1). Any eventual postoperative wound healing complications in the donor site were considered exclusion criteria.

### 2.2. Operative Procedure

After radial forearm free flap harvesting, the donor site was grafted with a split-thickness skin graft (STSG) taken from the anterolateral aspect of the right thigh of the patient, with the interposition of Matriderm (MedSkin Solutions DR. Suwelack AG, Billerbeck, Germany) for 6/11 of our patients. An electric dermatome regulated to 0.2 mm of thickness was used to raise the STSG, followed by a meshing step procedure with a ratio of 1.5:1. After appropriate preparation of the palmar forearm wound with sterile water rinsing, hemostasis with bipolar electrocautery, and human fibrin glue (Artiss, Baxter, Mississauga, ON, Canada) coverage, the STSG was carefully applied, outstretched and fixed with staples. A non-adhering silicone surface (Adaptic Touch, 3M, Rüschlikon, Switzerland) and gauze were used as a dressing, which was removed on postoperative day 5 if no complications were encountered.

### 2.3. Outcomes

**Figure 2:**
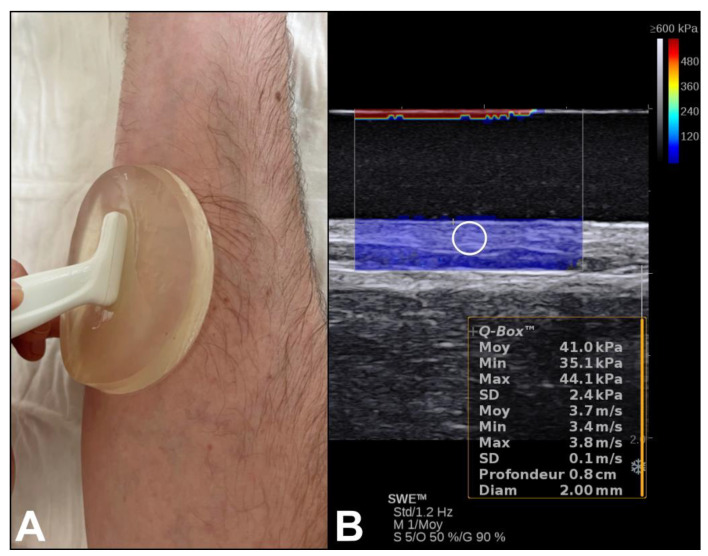
(**A**) Examination using a high-resolution 6–20 MHz linear array transducer with a thin-style gel pad. (**B**) The colored region represents the 2D quantitative elastogram superimposed on a B-mode image with a color scale (see top right). The software allowed us to measure the mean stiffness (Young’s modulus, in kPa) value and the shear wave velocity (m/s) of the skin inside a white circular region of interest (2 mm diameter Q-Box).

Donor site wound ultrasound examinations at forearm level (and at the same site on the contralateral forearm as control) were performed with the forearm and hand placed on a table in a supine and rest position in all included patients. Examinations were performed by two observers (S.D. and M.G.) on an Aixplorer™ ultrasound system (Supersonic Imagine, Aix-en-Provence, France). A high-resolution linear 6-20 MHz linear array transducer (SuperLinear™ 20-6 HockeyStick; Supersonic Imagine, Aix-en-Provence, France) with 192 elements and a bandwidth from 6 to 20 MHz was used. The protocol was standardized using a thin-style gel pad (Hill Laboratories company, Frazer, PA, USA). In B-mode, the skin’s morphology was evaluated, and SWE with a 2 mm diameter Q-Box (quantitative box) focus area was performed from the surface of the skin to a depth of 2 mm in the longitudinal and transverse plane in the operated site at the level of the forearm in the middle of the recipient site and at the same site on the contralateral forearm as control (Figure 2). The quantitative values of the elasticity of the skin were obtained in m/s (shear wave speed) and in kPa (shear elastic modulus).

Erythema and pigmentation quantification of the donor site wound and contralateral side was performed using Dermacatch^®^ (Colorix™, Neuchatel, Switzerland), and measurement of the degree of threshold sensitivity to an exerted force or pressure at the donor site wound and contralateral side was performed using the Semmes–Weinstein monofilament test.

Patients’ personal global (functional and aesthetical) satisfaction ratings and the subjective questionnaire of The Patient and Observer Scar Assessment Scale (POSAS) were recorded.

### 2.4. Statistical Analysis

In our study, the data spread was nonparametric; the Mann–Whitney was applied for all the statistical comparisons we performed. Significance was expressed as * *p*  <  0.05, ** *p*  <  0.01, and *** *p*  <  0.001. All analyses were performed using GraphPad Prism 8 (GraphPad Software, La Jolla, CA, USA).

## 3. Results

The study included 11 patients with a mean age of 36.3 years (SD 9.3 years). Four patients were smokers. Radial forearm free flap harvesting was performed on the right side for four patients and on the left side for seven patients. Comorbidity was observed in only one patient (asthma).

### 3.1. Shear Wave Elastography

**Figure 3:**
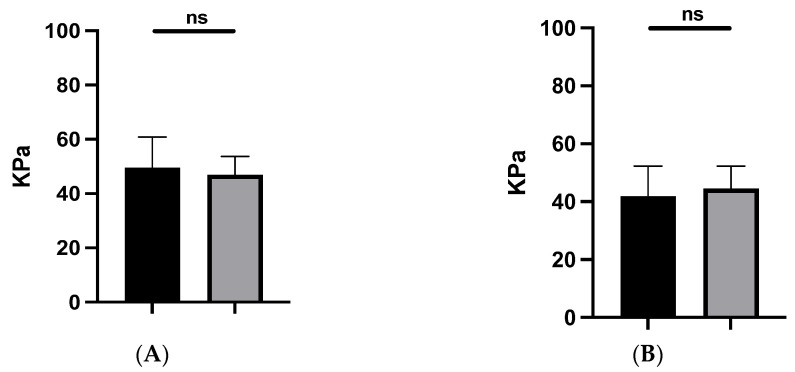
Shear wave modulus (KPa) of the skin in the transverse (**A**) and longitudinal (**B**) planes. Operated site (black) and contralateral site (grey). ns: not significant.

We did not find any significant difference in terms of Young’s modulus in both the longitudinal and transverse assessments. Indeed, our cohort patients evidenced similar mechanical properties in the transverse and longitudinal planes (*p* = 0.95 and *p* = 0.75, respectively) between the forearm skin graft and the contralateral non-operated side. (Figure 3). Similarly, the interposition of Matriderm in the surface between subcutaneous tissue/fascial tissue and the skin graft did not influence the stiffness of our sample compared to the non-operated side in the transverse and longitudinal planes (*p* = 0.15 and *p* = 0.35, respectively).

### 3.2. Coloration

**Figure 4:**
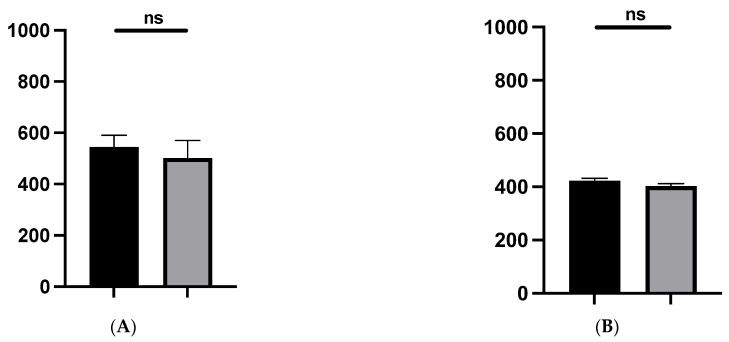
Pigmentation quantification (melanin) (**A**) and erythema (**B**) of the skin. Operated site (black) and contralateral site (grey). No notable difference in scar color, particularly in terms of inflammation (erythema) and melanin concentration, was observed between the operated (experimental) and non-operated (control) sides. ns: not significant.

Secondly, taking into account the color of the scar, particularly in terms of pigmentation quantification and inflammation (erythema), our findings (Figure 4) did not show any significant difference (*p* = 0.32 and *p* = 0.06, respectively) between operated group (operated side) and control (non-operated side).

### 3.3. Sensitivity

**Figure 5:**
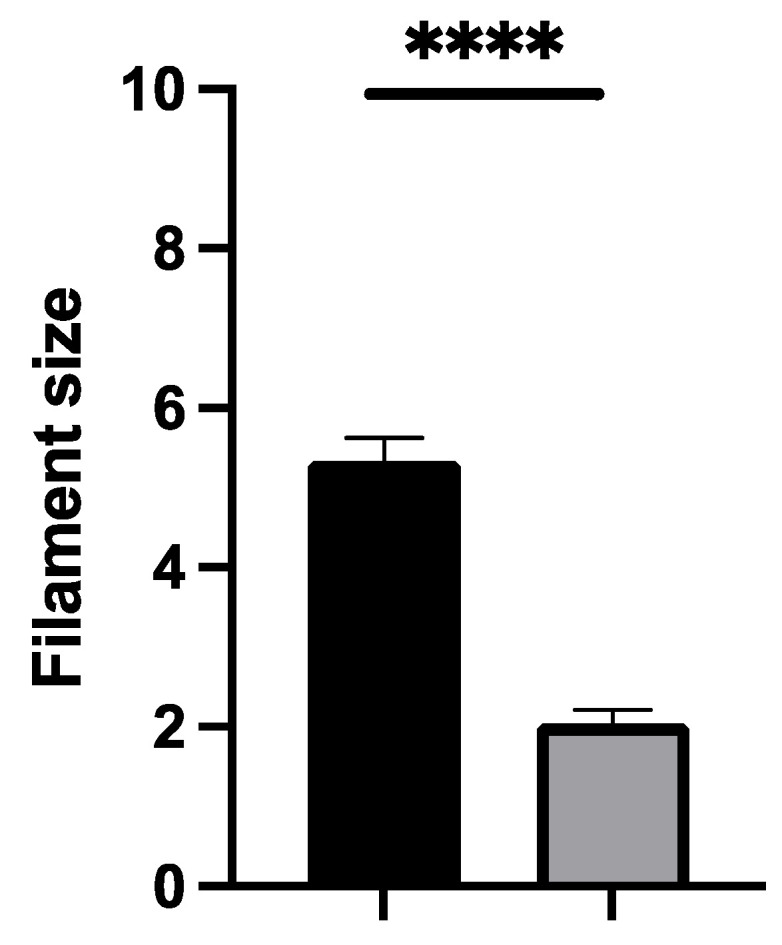
Semmes–Weinstein monofilament test. Operated site (black) and contralateral site (grey). Patients reported a significant decrease in sensation (loss of discrimination) when tested using the Semmes–Weinstein monofilament test. This indicates impaired tactile sensitivity at the scar site compared to the non-operated side. ****: significant.

Thirdly, considering the sensory outcomes, our patients significantly reported a loss of discrimination when tested by the Semmes–Weinstein monofilament test (Figure 5). There is a significant difference between the operated site and the contralateral side (*p* < 0.0001).

### 3.4. Subjective Scar Evaluation

Regarding the patients’ subjective scar evaluation (outlined by the POSAS questionnaire), we pointed out the following data: 91% of patients answered the questionnaire, but 100% were partially incomplete.

The results appeared in line with our quantitative and objective results. In detail, we observed that the majority of the patients (75%) did not report any scar pain or itching, they did not consider the scar discolored or dyschromic (100%), and they felt that the scar was of similar stiffness (80%), normo-trophic (83%), and without irregularity (83%) when compared to normal skin. Overall, their satisfaction was high: 83% of patients considered their scar “as normal skin”. One respondent rated the scar as moderately painful and itchy (Table 1).

## 4. Discussion

The use of high-frequency ultrasonographic transducers has made elastographic evaluation of the skin possible [19]. Most of the previous studies using elastography for skin assessment included patients with cancer, connective tissue disease, chronic systemic inflammation, lipodermatosclerosis, or risk of ulceration [20], but it may also find applications in aesthetic medicine. The recent literature underscores SWE’s potential in offering noninvasive and objective scar evaluation and suggests that it may be a promising tool for comprehensive surgical scar assessment. SWE distinguishes normal skin from scars and could be used to evaluate scar severity, which could be important for patient care and treatment. In burn scar evaluation, intra- and inter-observer reliability were excellent, even when performed by a novice clinician versus an experienced sonographer. A significant correlation was established between scar thickness, scar pliability, and SWS [21]. However, a mismatch between SWE and pliability scores in pathological and non-pathological scars in burn patients has also been reported [21]. Hang et al. tried to find a correlation between shear elastic modulus and the Vancouver scar scale indexes (VSSs), such as pliability, thickness, pigmentation, and pruritus. The association between stiffness and pliability was the highest, showing the existence of a direct correlation between all these parameters. However, the authors did not find a linear correlation, explained by the complexity of the wound healing process. They found a sort of “threshold effect”. This phenomenon is particularly true for hypertrophic scars [22]. Similar studies showed that hypertrophic scars slowly increase in stiffness over the first 6–12 months, then slowly regress over the following 2 years [23]. Non-pathological scars, on the other hand, may exhibit early induration, with stiffness decreasing to normal levels by 13 months post-injury [24]. Likewise, SWE was used to quantify keloid response to treatment after intralesional corticosteroid injection [25], with no significant difference in thickness between normal skin and treated keloids. SWE values of treated keloids were significantly lower but still higher than normal skin. Its ability to quantify tissue stiffness and elasticity can provide objective data for assessing scar biomechanics, offering a proactive approach to mitigate scar-related complications such as hypertrophic scarring or keloids.

Yang et al. [26] evaluated 60 healthy controls with SWE, showing a wide range of shear modulus for normal skin, ranging from 6.9 kPa in the abdomen to 43.8 kPa on the dorsal middle phalanx of the finger. The mean elastic modulus values were 30.3 kPa for the finger, 14.8 kPa for the forearm, 17.8 kPa for the chest wall, and 9.5 kPa for the abdominal wall, and reference ranges of normal skin elasticity were 12.1 to 48.4 kPa for the finger, 3.5 to 26.0 kPa for the forearm, 6.6 to 28.9 kPa for the chest wall, and 3.5 to 15.5 kPa for the abdominal wall. Men showed higher skin elasticity measurements than women, and elastic modulus appeared more elevated in participants aged 20 to 50 years than in the other groups. The body mass index and skin thickness had no significant impact on skin elasticity measurements [26]. Therefore, skin stiffness is known to vary with a number of variables, including different body locations [27], sex [26], and age [28], but also fiber orientation, collagen, and elastin fiber architectures [26]. At the same time, cutaneous aging is determined by intrinsic and extrinsic factors, such as dermal atrophy due to collagen loss, degeneration of the elastic fiber network, and loss of hydration [29,30,31]. Other studies reported that skin elastic modulus values were higher with reduced subcutaneous fat thickness. Consequently, the highest elastic modulus value found at the finger might be due to low subcutaneous fat thickness and the proximity to the bone; conversely, the abdominal wall had the lowest elastic modulus value because of higher fat deposition and no bone influence [32].

From a physiological perspective, the literature agrees about the absence of variation in terms of stiffness of contralateral healthy skin for the same symmetric sites: the variations in stiffness should derive only from the alterations determined by the pathological process. Similarly, Xiang et al. [19] confirmed that the elasticity of transverse and longitudinal sections significantly differed only on the dorsal middle finger, but for all the other anatomical sites explored, no significant differences were reported, in line with our findings. These findings provide evidence that contralateral skin elasticity can be used as a valid control for skin disease assessment.

Achieving optimal reconstructive results and minimizing donor site morbidity are the key factors in reconstructive surgery. While the free radial forearm flap is prized for its versatility and thin, pliable tissue, concerns persist regarding long-term aesthetic and functional consequences at the donor site, such as scar appearance, functional impact, patient satisfaction, and donor site complications [33]. Advancements in surgical techniques and postoperative care have been explored to mitigate complications and improve scar outcomes, such as flap thickness, wound closure methods, and rehabilitation protocols, which play a pivotal role in determining the outcome. Many studies emphasize the importance of patient-reported outcomes for a comprehensive assessment that goes beyond physical appearance [34]. The Patient and Observer Scar Assessment Scale (POSAS) has emerged as a widely utilized tool in the realm of surgical scar evaluation. Its significance in providing a standardized and comprehensive approach to assessing scars from both the patient’s and observer’s perspectives has been validated. One key strength of the POSAS lies in its dual assessment, encompassing patient-reported outcomes and objective observer evaluations [35,36,37].

In our study, the forearm donor site was grafted with a split-thickness skin graft from the thigh with the interposition of Matriderm (6 out of 11 cases, 5 out of 11 without Matriderm). We evaluated skin-grafted donor site scarring (with or without Matriderm) in terms of Young’s modulus, inflammation (erythema) and melanin concentration, sensory outcomes assessing the tactile discrimination by the Semmes–Weinstein monofilament test, and finally, the patients’ subjective scar evaluation outlined by the POSAS questionnaire.

Interestingly, inflammation and dyschromia of the skin graft were not significant when compared to the healthy site. The subjective patient satisfaction and scar perception evidenced that most patients did not experience scar-related pain or itching, patients did not perceive the scar as discolored or dyschromic, most patients considered the scar to have similar stiffness, a normal appearance, and no irregularities compared to normal skin. Unfortunately, despite 91% of patients answering the questionnaire, not all responses were fully completed. In our patient cohort, homogeneously distributed in terms of sex (all male-to-female patients) and age, we did not find any significant difference at a minimum of one year following surgery in terms of elastic modulus between the operated site and the contralateral one (*p* > 0.05), independently of Matriderm application. Unlike Yang et al., our range of stiffness at the forearm varied between 46.9 KPa and 69.8 KPa [26]. Considering the sensory outcomes, our patients significantly reported a loss of discrimination when tested by the Semmes–Weinstein monofilament test, but apparently, they are not affected in their everyday life as a large proportion of them (83%) consider their scar as “normal skin”.

Recently, Gierek et al. investigated a combination of acellular dermal matrix (ADM) with STSG to cover extensive defects after radical debridement in hidradenitis suppurativa [38]. They obtained significantly less inflamed scars, which were more elastic and less painful with satisfactory functional and aesthetic outcomes when evaluated by the Vancouver Scar Scale. Interestingly, among the different outcomes tested, the authors performed an indirect stiffness measurement using a cutometer to quantify the tissue deformity through the application of a suction/vacuum force to the upper layer of the skin. The aim was to determine the elasticity of the skin using a non-contact optical measuring system (cutometer). Previously, the co-graft ADM and STSG technique showed an interesting synergy even in donor site healing after a free flap, leading to significantly less painful and softer scars. Cosmetic outcomes were higher according to POSAS for both patients and surgeons [39].

### Limitations

Further concerns around reproducibility and standardization of SWE for skin lesions are related to the proximity between the probe and the skin itself. Subcutaneous tissues may cause overestimation or underestimation of the signal, resulting in a low signal-to-noise ratio. How subcutaneous fat, muscle, and bones influence the measurement is still not clear. Recent studies have mentioned a cut-off of 3 mm in subcutaneous fat thickness as discriminating between high and poor SWE reproducibility; since the elastic modulus of muscle and bone is apparently higher than that of skin within a 1 to 2 mm spatial resolution, the signal of muscle and bone may override the signal of skin within the range of 1 to 2 mm [32,40]. Finally, measurements of skin stiffness may be biased because of the inevitable use of ultrasound gel and compressive maneuvers [41]. In our study, measurements were performed exactly in the middle of the recipient site in the longitudinal and transverse planes, but SWE measurements can be affected by small changes in the position of the region of interest. We used the shear modulus in our analysis instead of SW velocity (SWS). Therefore, our results can be generalized and compared with the current literature. However, the algorithm used to calculate the shear modulus from the SWS has not received any approval for dermatological use at the moment. Furthermore, different machines, when evaluated under similar conditions, produced different SWS values, which consequently led to different SWE values [42].

In addition, our study has some limitations, including the small sample size as well as the single-center modality, and the retrospective nature which can lead to unavoidable bias in clinical outcomes. The nature of the scar is potentially different between one year and three years after surgery, but probably due to the small sample size, we have not found any correlation between time after surgery and measurement values. In addition, histological assessment was not performed since a skin biopsy is an invasive procedure and can worsen pathological scars. The subjective assessments such as the reliance on patient-reported outcomes, lack of standardized protocols for data collection and interpretation, and the missed inter-rater (different operators) and intra-rater (multiple measurements) reliability tests.

## 5. Conclusions

SWE is an accessible, objective, noninvasive imaging modality that is applied more often in clinical practice to measure stiffness in a variety of soft tissues and physiological or pathological processes such as skin aging, scars, systemic sclerosis, lymphoma, and other skin cancers. With the growing interest in developing new elastography applications, we showed a further application in wound healing. This technique has a promising value, but further significant investigation needs to be conducted to address the technical limitations concerning standardization and reproducibility.

## Figures and Tables

**Table 1:** Subjective scar evaluation (POSAS).

Scar pain or itching	25%
Scar discoloration or dyschromia	0%
Stiffness	20%
Scar dystrophy	17%
Scar irregularity	17%
Overall high satisfaction	83%

## Data Availability

The data that support the findings of this study are available on request from the corresponding author (SD). The data are not publicly available due to information that could compromise the privacy of research participants.
